# Supplementary material for: geneshot: gene-level metagenomics identifies genome islands associated with immunotherapy response
Source: Genome Biol. 2021 May 5;22:135. doi: 10.1186/s13059-021-02355-6 (PMC8097837; doi:10.1186/s13059-021-02355-6)
Supplement: Supplementary file 1 — Additional file 1: Figure S1. Summary of gene-level metagenomic analysis results generated by geneshot. The results for each specimen across all cohorts is shown in comparison to the number of sequence reads for each specimen (x-position) including the number of genes detected by de novo assembly of each individual specimen (A), the proportion of reads from each specimen which align uniquely to the de novo gene catalog (B), and the number of genes from the gene catalog detected by alignment (C). The distribution of genes across CAGs of different sizes is shown in (D), with the horizontal axis showing the CAG size range and the vertical axis showing the number of genes belonging to CAGs within that size range. (E) shows the relative abundance (log10) of the CAGs with the highest relative abundance (rows, annotated by majority taxonomic annotation) across all specimens (columns, annotated by BioProject and ICI response label), with hierarchical clustering by relative abundance across rows and columns. Figure S2. Visual depiction of CAG-level abundance calculation. Each CAG consists of a group of genes, each of which is detected in each specimen by alignment of WGS reads. The abundance of genes from each CAG are shown in (A) and (C) in units of the absolute number of reads aligned uniquely from each specimen. The ‘depth’ of sequencing of each gene is a commonly-used length-normalized measure in which the total number of bases from all aligned reads is divided by the length of each gene. Genes are grouped into CAGs based on average linkage clustering using the cosine distance metric computed from the vector of sequencing depth values across all specimens. The abundance of each CAG is computed as the sum of sequencing depth for all genes in the CAG, divided by the sum of sequencing depth of all genes detected. That proportional abundance metric is shown for each CAG in (B) and (D). Specimens are arranged in alphabetical order on the horizontal axis, with abundance measur [file 13059_2021_2355_MOESM1_ESM.docx]

**Supplementary Figures**


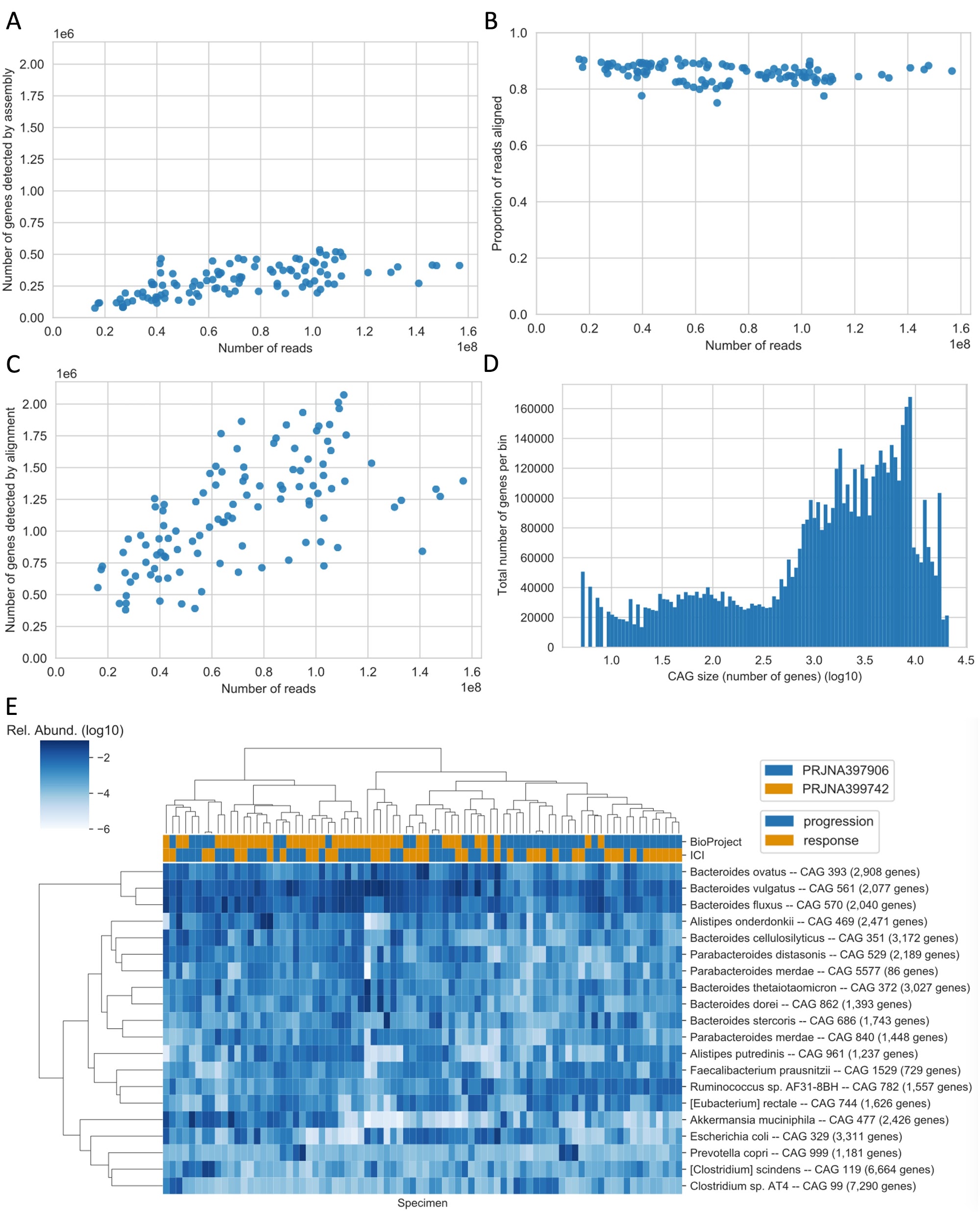


Fig S1. Summary of gene-level metagenomic analysis results generated by *geneshot*. The results for each specimen across all cohorts is shown in comparison to the number of sequence reads for each specimen (x-position) including the number of genes detected by *de novo* assembly of each individual specimen (A), the proportion of reads from each specimen which align uniquely to the *de novo* gene catalog (B), and the number of genes from the gene catalog detected by alignment (C). The distribution of genes across CAGs of different sizes is shown in (D), with the horizontal axis showing the CAG size range and the vertical axis showing the number of genes belonging to CAGs within that size range. (E) shows the relative abundance (log10) of the CAGs with the highest relative abundance (rows, annotated by majority taxonomic annotation) across all specimens (columns, annotated by BioProject and ICI response label), with hierarchical clustering by relative abundance across rows and columns.


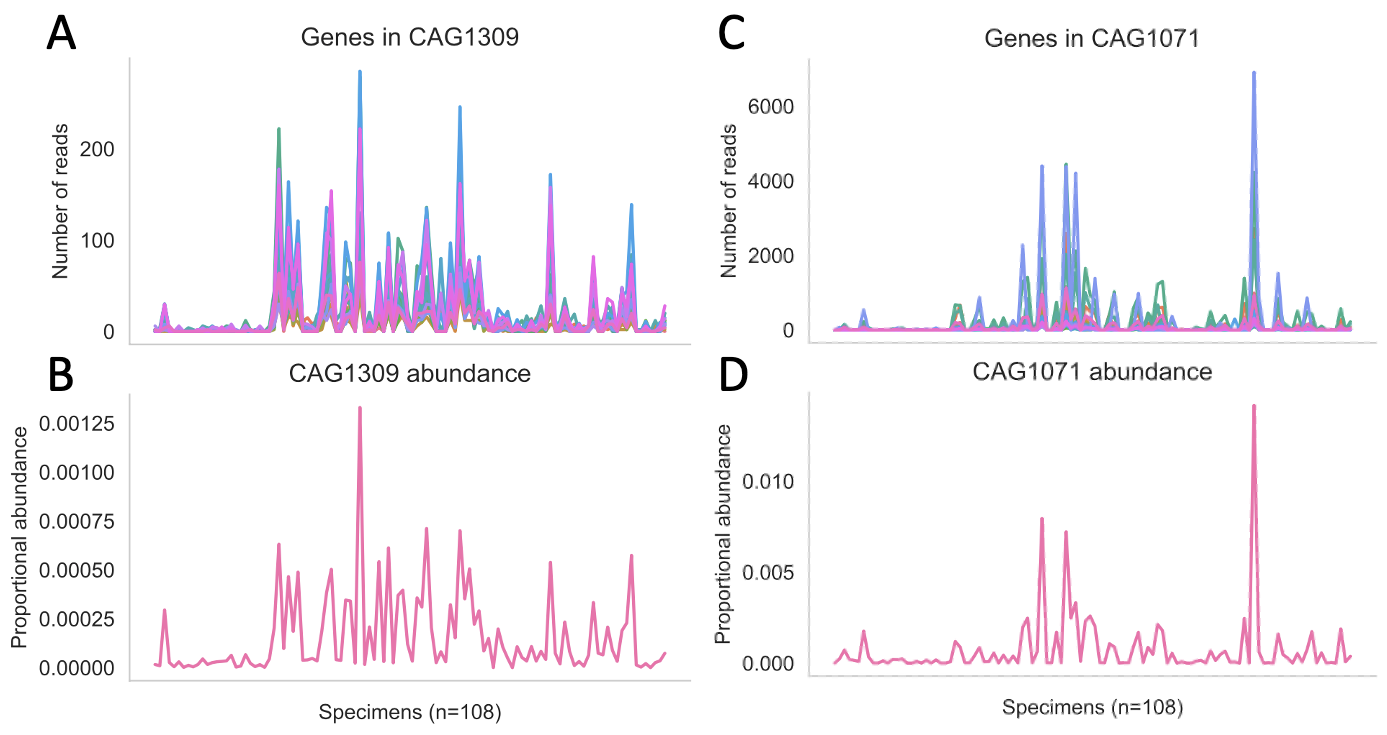


Fig S2. Visual depiction of CAG-level abundance calculation. Each CAG consists of a group of genes, each of which is detected in each specimen by alignment of WGS reads. The abundance of genes from each CAG are shown in (A) and (C) in units of the absolute number of reads aligned uniquely from each specimen. The ‘depth’ of sequencing of each gene is a commonly-used length-normalized measure in which the total number of bases from all aligned reads is divided by the length of each gene. Genes are grouped into CAGs based on average linkage clustering using the cosine distance metric computed from the vector of sequencing depth values across all specimens. The abundance of each CAG is computed as the sum of sequencing depth for all genes in the CAG, divided by the sum of sequencing depth of all genes detected. That proportional abundance metric is shown for each CAG in (B) and (D). Specimens are arranged in alphabetical order on the horizontal axis, with abundance measures for each specimen on the vertical axis.


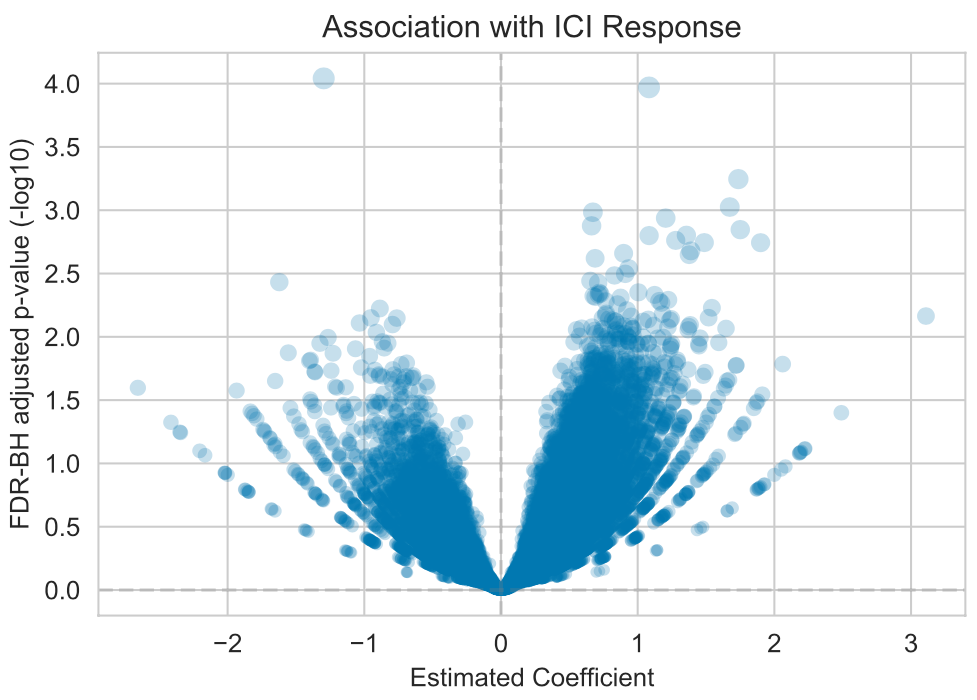


Fig S3. Volcano plot showing the distribution of association estimates for CAGs with ICI response. Each point represents a single CAG, showing all CAGs which contain at least five constituent genes. The horizontal axis shows the estimated coefficient of association with ICI response (positive values indicate a higher relative abundance in ICI responders in comparison to progressors). The vertical axis shows the *p*-value for that coefficient of association for each CAG after adjustment for multiple hypothesis testing with the FDR-BH procedure.


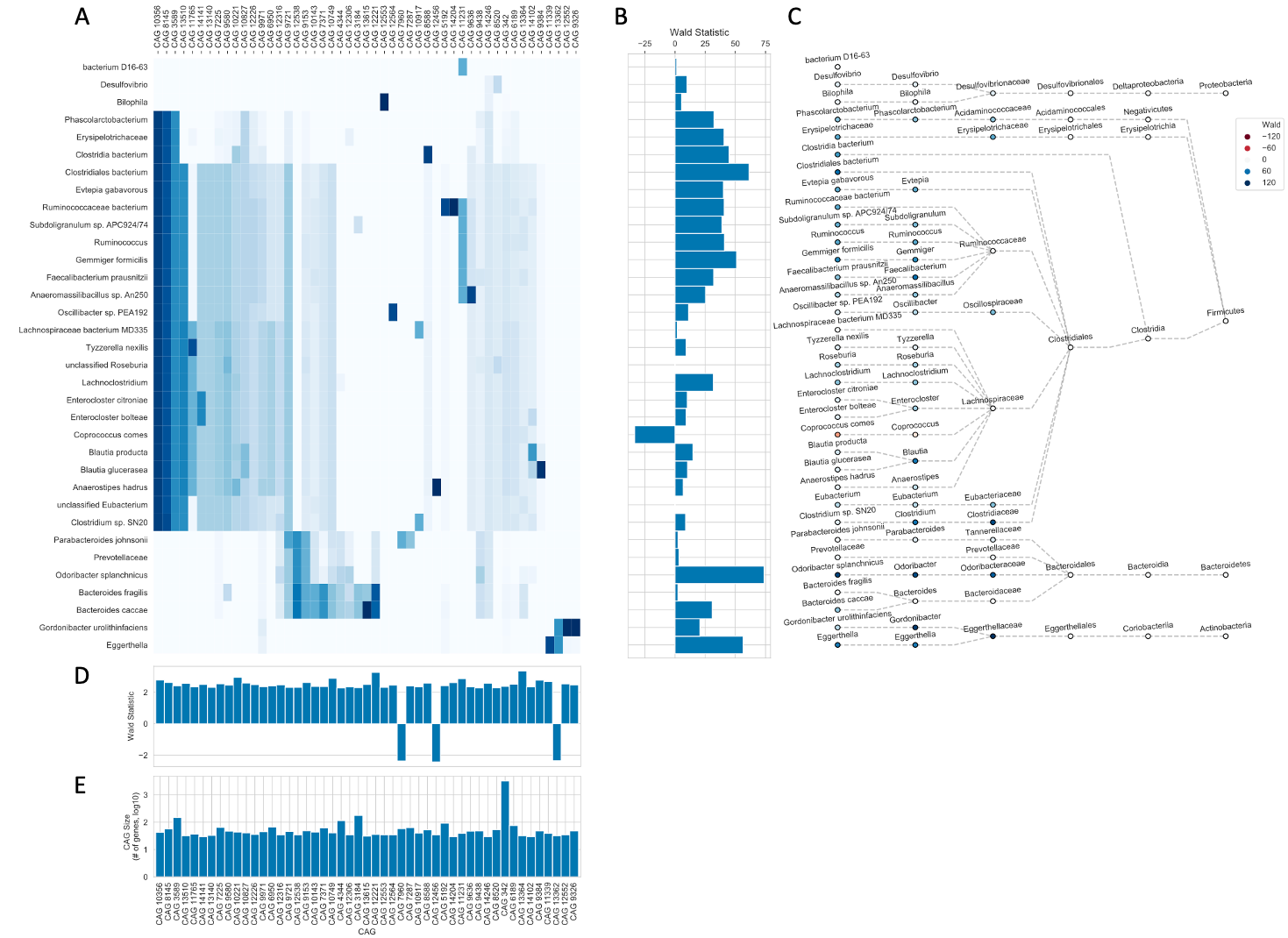


Fig S4. Taxonomic annotation of CAGs associated with ICI response. The taxonomic annotation of genes found in the 50 CAGs (columns) which were most consistently associated with ICI response (after applying a minimum size threshold of 30 genes), are shown in (A) with shading indicating the proportion of genes assigned to the indicated taxon (rows). The Wald statistic summarizing the association with ICI response is shown for taxa in (B) and for individual CAGs in (D). A taxonomic tree annotating those organisms is shown in (C), and CAG size is shown in (G).


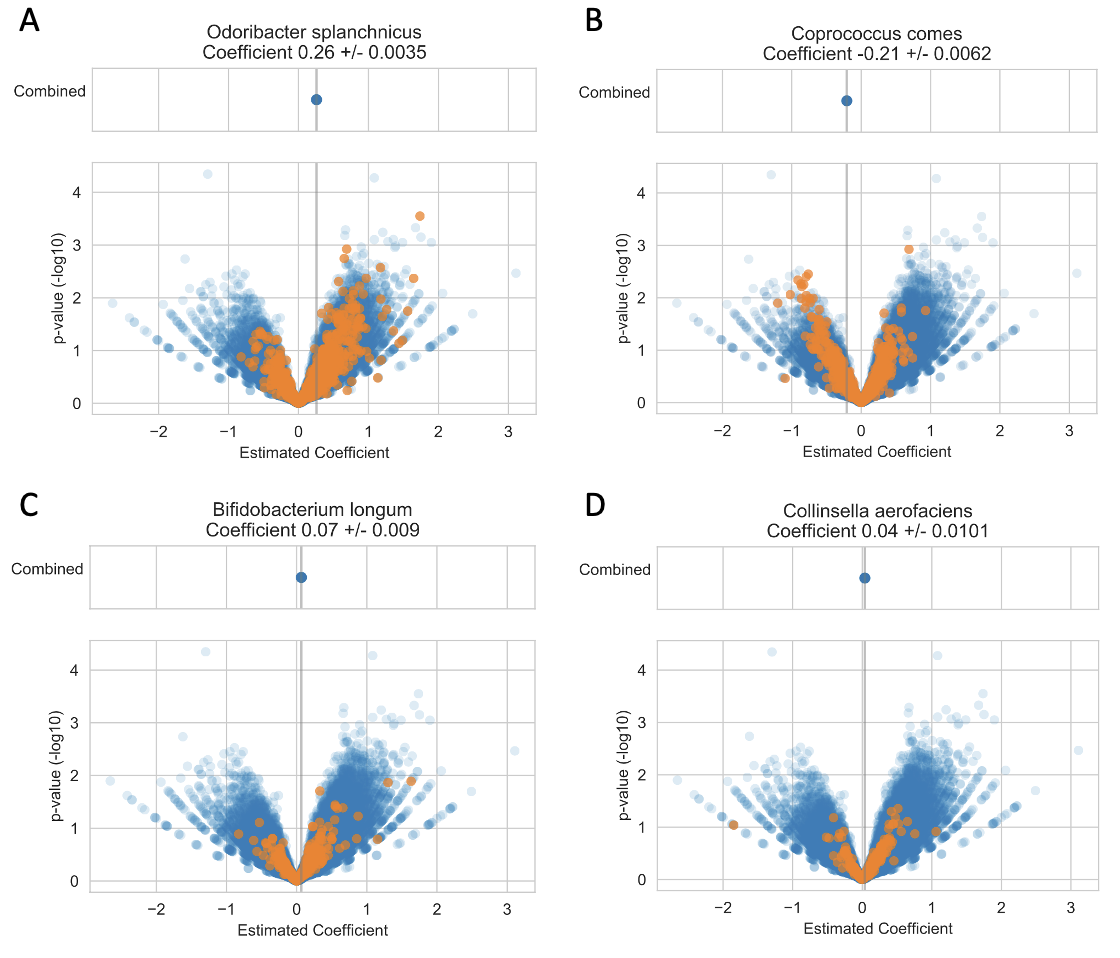


Fig S5. Visual depiction of the aggregation of CAG-level ICI-response association metrics by taxon. For each organism, the lower panel shows the estimated coefficient and -log10 *p*-value for the association of each CAG with ICI response. Every CAG either contains at least one gene which was taxonomically annotated to the indicated organism (orange), or there were no genes in that CAG with that taxonomic annotation (blue). The aggregate taxon-level ICI-response association for each organism is shown in the upper panel from each subplot, with the estimated coefficient +/- standard deviation indicated in the subplot title. The vertical grey line in each subplot marks the taxon-level estimated coefficient value.


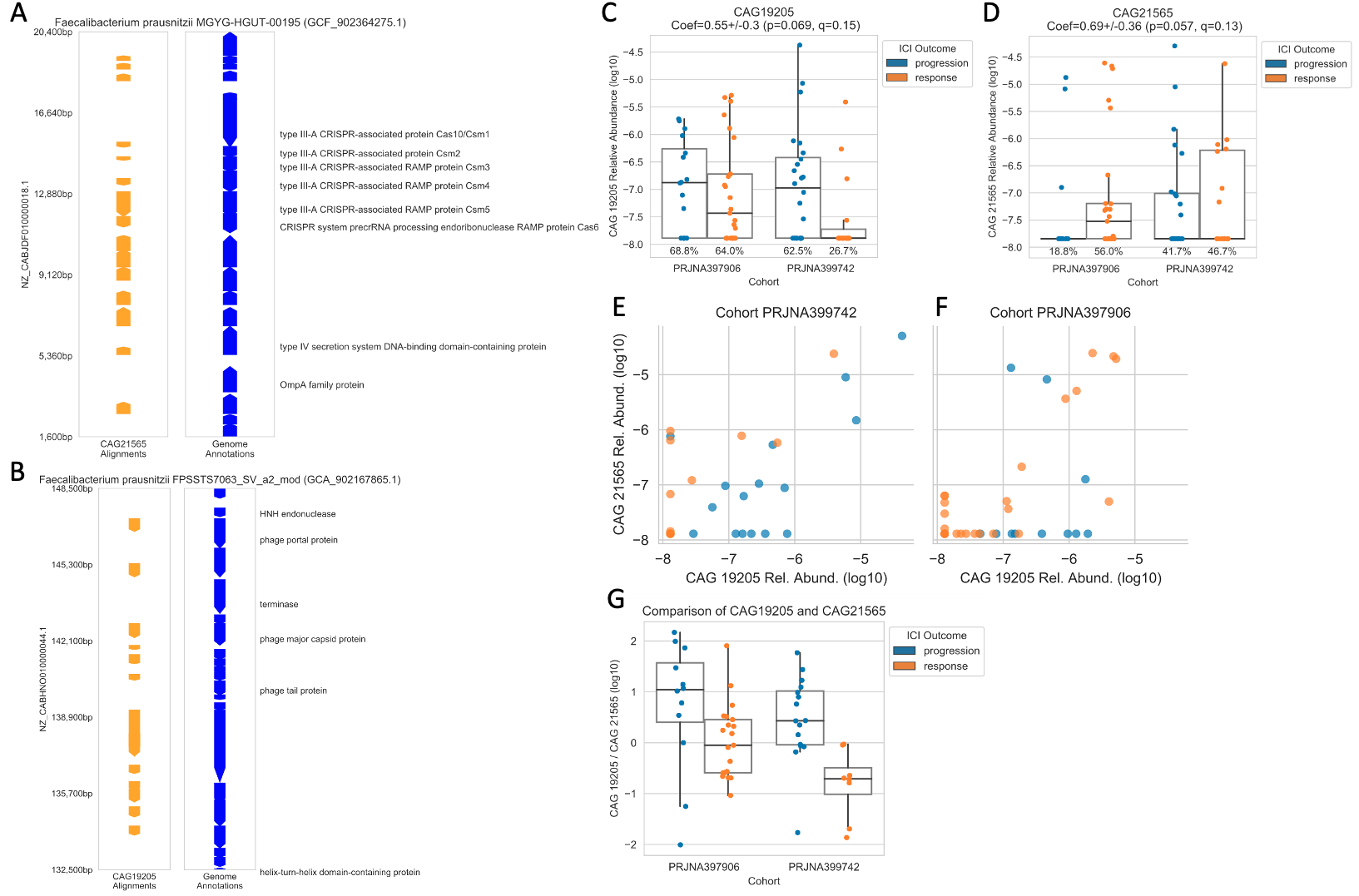


Fig S6. A putative bacteriophage of *Faecalibacterium prausnitzii* is associated with ICI non-response, in contrast to an associated bacteriophage defense island that is associated with ICI response. The genomic coordinates of ICI response-associated CAGs are shown for two strains of *F. prausnitzii* (as in Figure 2) (A-B). In addition, the relative abundance of these two CAGs is compared directly (C-F), and the ratio of relative abundances is shown as a function of ICI response across both cohorts (G).


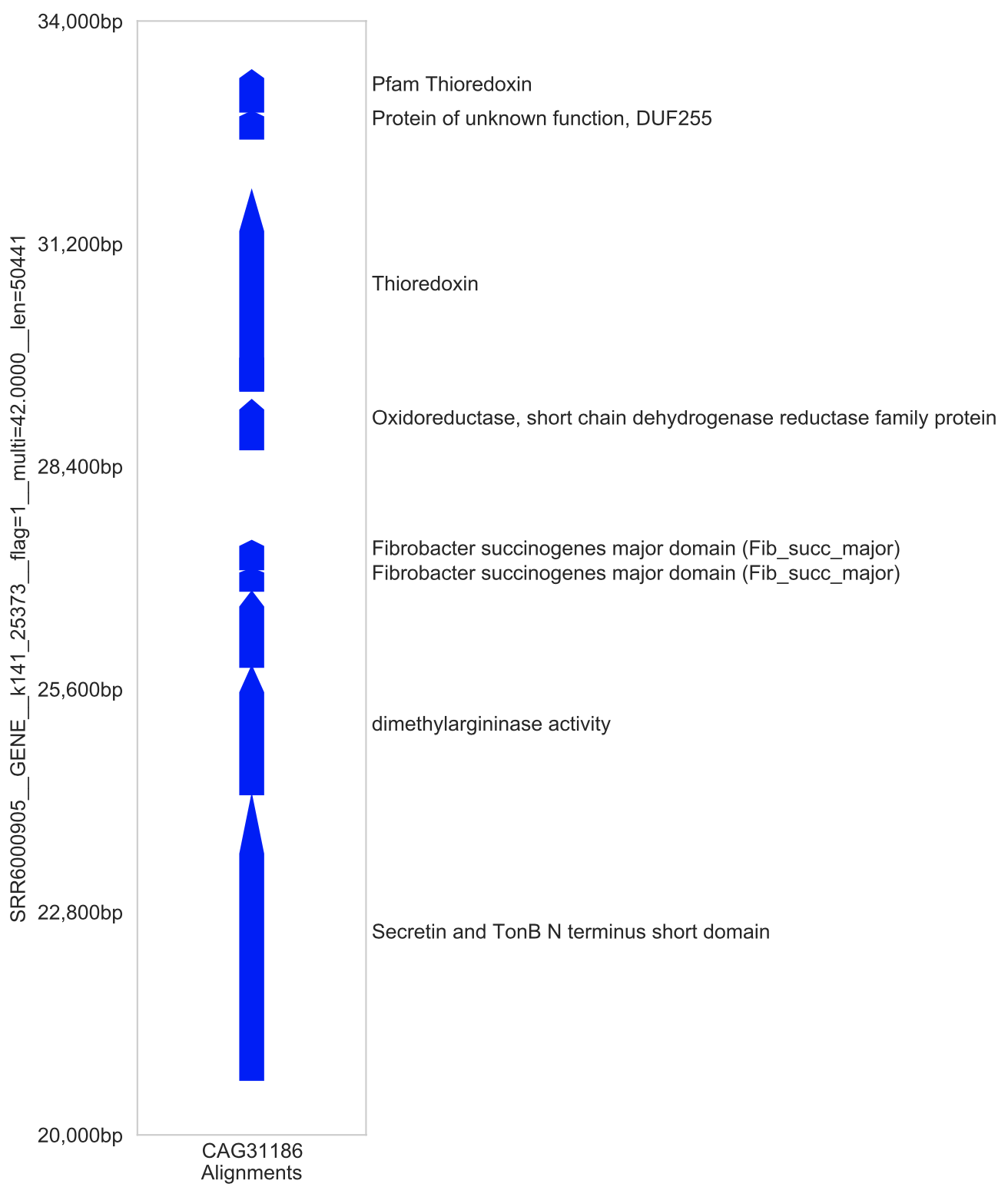


Fig S7. Example of CAG alignment to *de novo* assembly. The alignment coordinates for the alignment of a single CAG against the contigs generated by *de novo* assembly from this dataset is shown alongside the eggNOG functional annotations for the underlying genes. All alignments covered the entirety of the gene catalog sequence at >99% amino acid identity. The 14kb physical region shown in this plot encompasses all such high quality alignments for this CAG in this specimen.
