# Supplementary material for: geneshot: gene-level metagenomics identifies genome islands associated with immunotherapy response
Source: Genome Biol. 2021 May 5;22:135. doi: 10.1186/s13059-021-02355-6 (PMC8097837; doi:10.1186/s13059-021-02355-6)
Supplement: Supplementary file 4 — Additional file 4. Review history. [file 13059_2021_2355_MOESM4_ESM.docx]

Review History

**First round of review**

**Reviewer 1**

**Are you able to assess all statistics in the manuscript, including the appropriateness of statistical tests used?**

I was able to assess all statistics in the manuscript, including the appropriateness of statistical tests used. No further review is recommended.

**Comments to author:**

- Are the methods appropriate to the aims of the study, are they well described, and are necessary controls included?

The methods are well defined. The tool presented here has really great documentation. We were able to download, run and test the tool presented here - and it seems to work with ease with only a few NextFlow related hiccups.

- Are the conclusions adequately supported by the data shown?

The conclusions are adequately supported by the data shown. This approach has proved helpful when studying some disease systems and will be widely applicable as the use of assembly-based approaches become more computationally accessible (which this does!)

- Are sufficient details provided to allow replication and comparison with related analyses that may have been performed?

Yes. Although we didn't try to replicate the study presented here, we were able to use the tool with ease.

- Is the method likely to be of broad utility? Is any software component easy to install and use? Please indicate briefly the novel features and/or advantages of the method, and/or please reference the relevant publications and which methods, if any, it should be compared with.

This tool will definitely help the community. By wrapping the tool in a workflow manager, like NextFlow, it will make it accessible to many more users that it otherwise would. The built in statistics are appropriate for the type of data generated in sequencing.

- Is the paper of broad interest to others in the field, or of outstanding interest to a broad audience of biologists?

Definitely! This paper will prove to be helpful for many in the field.

My only suggestion is to update the summary figure to better represent when data from a single sample is used and when that data is then merged. The tool presented here is statistically sound and a good approach to this type of data. The documentation is some of the best I've seen and I would be excited to use this on data!

**Reviewer 2**

**Are you able to assess all statistics in the manuscript, including the appropriateness of statistical tests used?**

Sound and appropriate statistics.

**Comments to author:**

The authors describe geneshot, an end-to-end microbiome analysis workflow (assembly and gene prediction, gene clustering info CAGs, taxonomic and functional annotations). While I did not have the chance to in-depth assess the software and results myself, other than making sure it executes on a smaller dataset I had floating around, the underlying methods are clearly described (and motivated) in the manuscript (and the supplement); results are clearly presented and - lacking proper standards (e.g. CAMI) to assess gene-level data - benchmarked just fine; the workflow itself is implemented using the improved Nextflow DSL-2 notation, tested and properly documented in the GitHub's wiki - including a getting started section -; and the authors quickly respond to (and resolve) problems via GitHub's issue tracker. This is reassuring, to say the least, and in fact exemplary. Hopefully this spirit stays alive after publication, which I wholeheartedly recommend *as is*.

**Response to Reviewers**

We thank the reviewers for taking the time to review our manuscript, as well as taking the additional time and effort to review the underlying software and run it on their own datasets. The positive response of the reviewers to both the content of the geneshot pipeline, as well as the implementation within the Nextflow workflow management system, helps provide us with the motivation to continue supporting the broad public utilization of this software resource for the microbiome research community. In doing so we recognize the importance of maintaining the documentation and responsiveness to submitted issues, as suggested by reviewer #1.

Regarding the suggestion to clarify the summary figure, we have modified the legend of Figure 1 to explicitly state which steps are executed on individual specimens, and which steps are aggregated across all specimens. This is an important nuance of the workflow, and we feel that adding this detail will significantly improve our ability to communicate the behavior of the software to all readers.
